# The effectiveness of the appropriate prophylactic antibiotic use program for surgery

**DOI:** 10.1017/ash.2023.233

**Published:** 2023-09-29

**Authors:** Eunjung Lee, Tae Hyong Kim, Se Yoon Park, Jongtak Jung, Yae Jee Baek

## Abstract

**Background:** Evaluation of the adequacy of prophylactic antibiotics in surgery has been implemented as a national policy in Korea since August 2007, and the appropriate use of prophylactic antibiotics has improved. However, antibiotic prescriptions that are not recommended or discontinuation of prophylactic antibiotic administration within 24 hours after surgery are still not well done. This study introduced a program to improve the adequacy of prophylactic antibiotics for surgery and analyzed its effects. **Methods:** We retrospectively analyzed the effectiveness of the appropriate prophylactic antibiotic use program for surgery conducted at a university hospital in Seoul. The participants were patients aged ≥18 years who underwent any of 18 types of surgery. The program started was implemented in June 2020. First, a computer system was used to confirm the antibiotic prescription recommended for each surgery. It also assessed whether the number of days of administration was exceeded, whether antibiotics were prescribed in combination, and whether antibiotics prescribed for discharge medicine were checked in 4 steps. A pop-up window appeared in each patient record to enter the reason for the prescription. If the reason was appropriate, the prescription was allowed, but if not, the prescription was restricted. In addition, infectious diseases physicians and an insurance review team visited each department to conduct an education session. To analyze the effect 3 months before activity (January–March 2020) and 3 months after activity (October–December 2020), we compared the first antibiotic administration rate within 1 hour prior to skin incision, the recommended prophylactic antibiotic administration rate, and surgery type. The rate of discontinuation of prophylactic antibiotics within 24 hours after administration and the rate of prescription of prophylactic antibiotics at discharge were compared. **Results:** In total, 1,339 surgeries during the study period were included in the analysis. There were 695 cases before the introduction of the program and 644 cases after the introduction. The rate of first antibiotic use within 1 hour prior to skin incision was 93.1%–99.5% (*P* < .001), the rate of recommended prophylactic antibiotic administration was 85.0%–99.2% (*P* < .001), and the rate of discontinuation of antibiotic administration within 24 hours after surgery improved from 51.8% to 98.3% (*P* < .001), respectively. The prescription rate of antibiotics at discharge improved from 20.7% to 0.8% (*P* <.001) (Table 1). **Conclusions:** A computerized program to improve the adequacy of prophylactic antibiotic use in surgery combined with education of medical staff was very effective.

**Figure:**
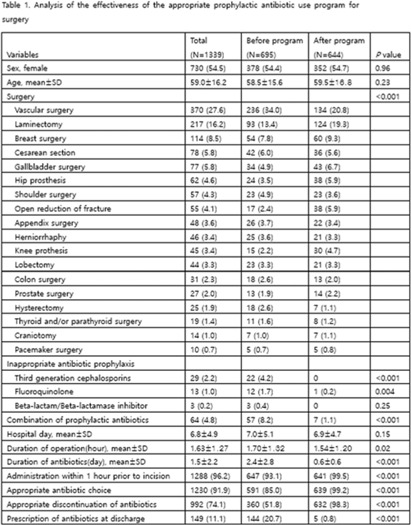

**Disclosure:** None

